# Intraepithelial lymphocytes, scores, mimickers and challenges in diagnosing gluten-sensitive enteropathy (celiac disease)

**DOI:** 10.3748/wjg.v23.i4.573

**Published:** 2017-01-28

**Authors:** Consolato Sergi, Fan Shen, Gerd Bouma

**Affiliations:** Consolato Sergi, TianYou Hospital, Wuhan University of Science and Technology, Wuhan 430081, Hubei Province, China; Consolato Sergi, Fan Shen, Departments of Laboratory Medicine and Pathology, University of Alberta, Edmonton AB T6G 2B7, Canada; Gerd Bouma, Department of Gastroenterology and Hepatology, Vrije Universiteit Medical Center, 1081 HV Amsterdam, The Netherlands

**Keywords:** Gluten, Duodenum, Lymphocytes

## Abstract

The upper digestive tract is routinely scoped for several causes of malabsorption, and the number of duodenal biopsy specimens has increased notably in the last 10 years. Gluten-sensitive enteropathy (GSE) is an autoimmune disease, which shows an increasing prevalence worldwide and requires a joint clinico-pathological approach. The classical histopathology of GSE with partial or total villous blunting is well recognized, but the classification of GSE is not straightforward. Moreover, several mimickers of GSE with intraepithelial lymphocytosis have been identified in the last 20 years, with drug interactions and medical comorbidities adding to the conundrum. In this review, we report on the normal duodenal mucosa, the clinical presentation and laboratory diagnosis of GSE, the duodenal intraepithelial lymphocytes and immunophenotype of GSE-associated lymphocytes, the GSE mimickers, the differences “across oceans” among guidelines in diagnosing GSE, and the use of a synoptic report for reporting duodenal biopsies in both children and adults in the 21^st^ century.

**Core tip:** Striking and unique microphotographs with comparison of classification of gluten-sensitive enteropathy across oceans and tables useful for the practice of gastroenterology.

## INTRODUCTION

In the last decade, there have been a plethora of publications in refining several gastrointestinal diseases. It has emerged as a period of an unceasing interest, particularly for diseases of the upper gastrointestinal tract[1-14]. In the hands of physicians reading histopathology reports, the number of duodenal biopsies with normal or near normal villous architecture and increased intraepithelial lymphocytes (IELs) appears to be collectively growing. Increased IELs or intraepithelial lymphocytosis in an otherwise apparently normal villous architecture can be a puzzle for both pathologists and treating physicians and may raise several differential diagnoses[15]. Moreover, the histopathology report may not be useful as it is, if it is not complemented with clinical and laboratory information. The report may be unsatisfactory due to lack of knowledge, incomplete performance of special stains or inadequate application of technical or professional skills. One or more of these issues may contribute to miscommunication between pathologists and clinical colleagues.

At first glance, gluten-sensitive enteropathy (GSE) or celiac disease seems to be straightforward, but it is not, neither from the clinical nor the pathologic point of view. There are mysteries behind this disease. Both the pathology and the pathogenesis are not yet fully unveiled. A few years ago, the European Society of Pediatric Gastroenterology, Hepatology and Nutrition (ESPGHAN) issued some guidelines on GSE, defining it as an “immune-mediated systemic disorder, elicited by gluten and related prolamins in genetically susceptible individuals”[16]. GSE was first described around 200 AD and is known in some countries as “sprue”, recalling the 18^th^ century-old Samuel Gee’s work “*On the Coeliac Affection*”[17].

To date, genetic studies have identified 43 predisposing loci that collectively explain some 50% of the genetic variance in GSE, but more than 90% of GSE-associated single nucleotide polymorphisms (SNPs) localize to the non-coding genome[18-20]. There is indeed a large epigenomic component that may play a contributive role, and a better understanding of the genomic-epigenomic relationship may be needed to translate genetic knowledge into future clinical practice[21,22]. In the meantime, pathology remains key in the diagnostic procedures and this review is composed of six parts, focusing on (1) the composition of normal duodenal mucosa; (2) the clinical presentation and laboratory diagnosis of GSE; (3) the immunophenotype of GSE-associated lymphocytes; (4) the GSE mimickers; (5) the differences “across oceans” among guidelines for diagnosing GSE; and (6) the use of a synoptic report for reporting duodenal biopsies in both children and adults.

## NORMAL DUODENAL MUCOSA

The villous character of the small bowel is intrinsically linked to the aim of an organism to increase its absorptive surface area. In early embryogenesis, development of the duodenal epithelium takes place from simple endodermal tubules between the 9^th^ and 10^th^ wk of gestation, when the epithelium converts to simple columnar epithelium. The epithelium ends its differentiation just 4-5 d before birth[23]. The usual configuration of the duodenal mucosa contains slender structures protruding from the surface, with 3-5 times the height of the crypts. The patchiness of the lymphoid nodules or mucosa-associated lymphatic tissue (MALT) needs to be considered in assessing the duodenal histology and can constitute one of the first pitfalls in interpreting a small intestinal biopsy.

According to our more than 20 years’ experience of reading duodenal biopsies of healthy individuals across ages, we can state that only very few lymphocytes can be usually seen among the epithelial cells. However, the IELs may vary during life and possibly in a circadian cycle. The IELs usually do not go over 5-10 per 100 epithelial cells in healthy individuals. The cut-off between pathological and normal has been decreased in the last three decades from 40 to either 20 or 25 lymphocytes per 100 epithelial cells[24]. Between 5-10 and the pathological threshold (20 or 25), there is a gap that has probably been inadequately investigated. The unveiled and/or underlying causes of the “near normal” cases (5-20 IELs/100 epithelial cells) may be intriguing. The presence of scattered normal lymphocytes in the surface epithelium of the duodenum is not well understood, although the prominent role of the duodenum in assessing the epitopes present in the food should be considered.

MALT of the gut is, indeed, crucial for the immunology and preservation of the microbiome[1,18,25]. Lymphocytes are recognized in the duodenal surface epithelium, because of some characteristics that allow them to be differentiated from the epithelial cells. Lymphocytes are characterized by their roundness, cell hyperchromasia, high nucleus-to-cytoplasm ratio, and quite constant intercellular distribution. However, the counting may be jeopardized by a number of factors, including the intrinsic and extrinsic conditions of biopsy grasping by the endoscopist, the laboratory processing of the tissue biopsy, and the individual evaluation of the pathologist[26-28].

Processing of a duodenal biopsy may represent a challenge for some laboratories. In fact, tangentially cut villi of appropriate duodenal regions may look like blunted and, thus, these areas should be avoided when an assessment of the villous architecture is crucial. Although criteria of adequacy are variable among authors of excellent reviews[29,30], in our opinion, it is desirable that biopsies containing at least 5 consecutive, intact villi that are well-oriented in the plane of section are sent for assessment to the pathologist. The conditions of “consecutiveness” and “intactness” are extremely important for the standardization of studies involving duodenal mucosal tissue. The correct orientation of the biopsy before paraffin embedding may be crucial and 3, but preferably 5 consecutive, intact and well-oriented villi are the minimum to adequately evaluate the villous architecture. In our opinion, if at least 5 villi are not consecutive, the diagnosis may be uncertain.

IELs are usually localized at the base of the surface epithelium in biopsies from healthy individuals. If the IELs are slightly increased in number, they tend to arrange themselves generally throughout the full thickness of the epithelium. Some ancillary studies have been proposed to give an accurate value of the lymphocytosis. Immunohistochemical typing of the cells has been suggested as an ancillary technique, but the normal upper limit has been suggested to be set higher than normal, at 29 IELs instead of 25 (or 24 instead of 20) per 100 epithelial cells[31]. The rationale for it is not fully clear, but values between 26 and 29 CD3-positive IELs may be empirically stated as borderline IEL. The term IEL should be reserved when IELs are equal to 30 or superior to this value. Duodenal portions may be different in the number of IELs present and this may also vary according to the ingested food.

Duodenal bulb biopsies may show more IELs than distal portions, and the other portions of the small bowel are also quite different from duodenum. In fact, villi of the distal bowel tend to be slightly taller, apart from areas overlying lymphoid aggregates, where they acquire broad based or flat shape. Underneath the surface epithelium, the lamina propria of the small bowel contains typically an infiltrate of scattered or mildly dense lymphocytes, plasma cells and eosinophils, which constitute the usual complement of the small bowel wall. The number of these three cell types in the lamina propria varies, but usually they are low in number. All three types can be easily recognized in normal biopsies and highlighted by immunohistochemistry or histochemistry using antibodies against CD3 (lymphocytes), CD138 (plasma cells) and Luna special stain (eosinophils), respectively. The presence of more than occasional plasma cells and more than 30 eosinophils per high power field (ocular × 10 and objective × 40) should be considered anomalous[28].

## CLINICAL PRESENTATION AND LABORATORY DIAGNOSIS OF GSE

**Figure 1:**
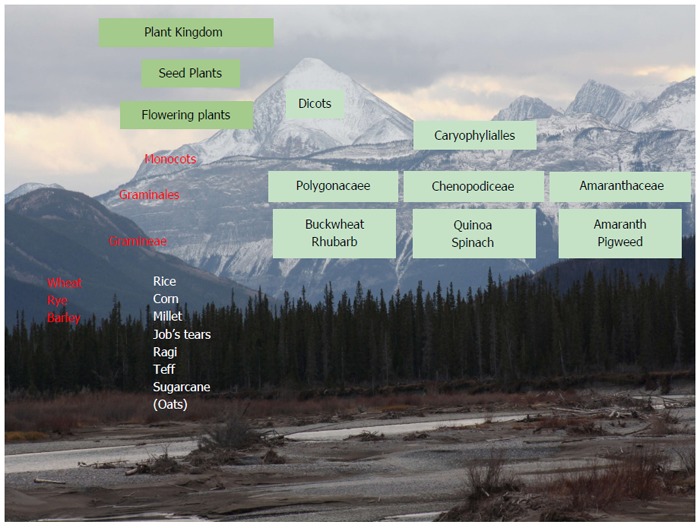
Plant taxonomy and celiac toxicity (red).

GSE is a dysregulation of the genome-epigenome, with abnormal reaction of the body to gluten-containing food[2,32-40]. In Figure 1, the plant taxonomy and celiac toxicity are depicted. Gluten is a protein that gives dough its elasticity, allowing it to rise without collapsing while trapping the CO_2_. It is important to remember that gluten is within wheat, rye and barley, but also in wheat derivatives: bulgar, couscous, mataza, seitan, semolina, triticale, spelt, kamut, einkorn, emmer and anything with “wheat” in the name (except buckwheat) as well.

The presentation of GSE in childhood is quite protean, including abdominal distension, diarrhoea, anorexia, weight loss, dermatitis herpetiformis and irritability. Conversely, the presentation of GSE in adulthood includes usually abdominal distension, steatorrhea, oedema and lethargy. Infrequent ways of presentation have been described in the literature[18,19]. In case of a suspicion of GSE, both children and adults are first screened using serologic studies for autoantibodies including IgA anti-tissue transglutaminase (aTTG)[35,41]. IgA anti-endomysial autoantibodies (EMA) have been previously used. IgA aTTG antibodies is the preferred test worldwide, showing a sensitivity of 94% and specificity of 97%[42,43]. Both tests are useful in IgA competent patients. The institution of a gluten-free diet (GFD) starts the decline of the titres of aTTG.

False-negative aTTG results may be seen in patients harbouring IgA deficiency, which is detected in 1/10 of the GSE patients and testing of IgG isotype of aTTG is mandatory. To date, deamidated gliadin peptide (DGP) seems to have better sensitivity in detecting early-stage GSE as compared to the TTG and EMA, and has been proposed to be the first line of investigation in IgA deficient patients (see below for differences among gastroenterological societies)[25,41,44,45]. False-positive results may occur in the setting of patients suffering from inflammatory bowel disease (IBD), primary biliary cirrhosis, cardiovascular disease, autoimmune enteropathy and other immune-mediated disorders[25,29,41,44,46]. Clinical correlation is paramount.

In the absence of supportive histologic or laboratory findings, a strong clinical suspicion should be followed by evaluation of high-susceptibility alleles of the human leukocyte antigens (HLA), HLA-DQ2 or DQ8, or repeating testing before starting gluten withdrawal[47]. If endoscopy is performed, the gross findings remain quite nonspecific and very subjective according to the experience of the endoscopist[48,49]. It is essential to not rely exclusively on the endoscopy, because up to 43% of paediatric patients harbouring GSE may show normal-appearing mucosa[50]. Thus, endoscopy should be almost exclusively associated with biopsy. The histologic examination of duodenal biopsies should be performed by a pathologist with specific skills in reading gastrointestinal biopsies. The routine collection of oesophageal and gastric biopsy specimens during upper endoscopy should be considered mandatory, particularly to clarify if gastritis or eosinophilic esophagitis are present.

In 2017, the gold standard for the diagnosis of GSE remains the tissue biopsy obtained at endoscopy. The histology of GSE relies on villous atrophy, IEL with or without enterocyte damage, increased inflammatory cells in the lamina propria and crypt hyperplasia[50,51]. If we consider the normal upper limit of 20 IELs or 25 IELs per 100 epithelial cells in sections stained with haematoxylin and eosin (HE), the normal ratio of IELs to epithelial cells is 1:5 or 1:4, respectively[2,31,36,52,53]. The presence of neutrophils is a common finding in the histopathology of GSE[54]. Neutrophilic infiltration of the lamina propria may occur in up to 1/3 of patients with GSE. Conversely, neutrophilic infiltration of the surface epithelium is seen more rarely.

Neutrophilic crypt abscesses are usually not seen in GSE, but are frequently seen in GSE mimickers, such as patients with infection, peptic duodenitis or autoimmune enteropathy (see below). The sensitivity of crypt hyperplasia may harbour a higher inter-individual variability and may not be obvious in some patients. Enterocyte damage, as defined by increased nuclear size and decreased cytoplasmic volume, and increased inflammation of the lamina propria, including increased numbers of lymphocytes and plasma cells in the lamina propria, are neither specific nor sensitive for GSE. These features are usually present in biopsies of different aetiology with marked abnormality of the mucosal architecture[54].

Eosinophils are not uncommon in the gastrointestinal tract[28,55]. Eosinophilic infiltration of the lamina propria may constitute a separate subgroup of GSE and it has been suggested to report clearly their presence only if sheets of eosinophils are seen. There is an association with eosinophils in the small bowel and oesophageal mucosa that has been increasingly recognized[28,56-58] and may require individualized assessment and treatment[30,59,60].

The presence of all histologic components makes the diagnosis of GSE certain indeed, but GSE may show only some of these features and some classifications have been proposed. The Oberhuber et al[61] modification of the original classification proposed by Marsh and Crowe remains a cornerstone for both pathologists and clinicians. Marsh classification identifies type I as an infiltrative lesion, characterized by IEL and a normal villous architecture of the duodenal mucosa, type II as an hyperplastic lesion, characterized by IEL and crypt hyperplasia and a normal villous architecture, type III as a destructive lesion, characterized by IEL, crypt hyperplasia and villous atrophy, and type IV as a hypoplastic lesion, characterized by a normal IEL count, normal crypt length and villous atrophy[62,63].

Oberhuber et al[61] modified the Marsh classification by dividing the type III lesions into three subtypes, including A (*alike* = near normal) or mild villous atrophy, B (*broad villi*) or marked villous atrophy, and C (*complete*) or completely flat mucosa[61,62] (Table 1). Corazza and Villanacci proposed to keep the type I infiltrative lesion with a setting of an upper limit of 25 IELs per 100 enterocytes[64] (Table 2). The type II hyperplastic lesion is rarely seen, while Oberhuber types IIIA and IIIB are grouped into a single category or grade B1. Corazza-Villanacci’s argument is pointing to the extreme variability between the same pathologist and different pathologists carrying a kappa divergence that is not minimal. Oberhuber stage IIIC is maintained in the revised classification as grade B2. Marsh-Oberhuber’s type IV hypoplastic lesion may now be considered obsolete.

**Table 1:** Revised Updated Marsh-Oberhuber classification of gluten-sensitive enteropathy (5 states of submucosal injury 0-4)

Type 0
Pre-infiltrative: normal V:C ratio and crypts with < 20-25 IELs per 100 enterocytes (1:5 or 1:4)
Type 1
Infiltrative type: normal V:C ratio and crypts, but ↑ IELs (≥ 20-25 IELs/100 enterocytes)
Type 2
Infiltrative-hyperplastic type: normal V:C ratio, but crypt hyperplasia with ↑ IELs
Type 3
Destructive (flat mucosa) type of GSE lesion according to the degree of villous atrophy
Type 3a: mild villous atrophy with V: C < 3:1, and ↑ IELs
Type 3b: marked villous atrophy with V: C < 1:1, and ↑ IELs
Type 3c: total villous atrophy with completely flat mucosa and ↑ IELs
Type 4
Atrophic type (hypoplastic); flat mucosa with only a few crypts and near-normal IEL count

V:C: Villous to crypt ratio (normal, V:C > 3:1); GSE: Gluten-sensitive enteropathy; IEL: Intraepithelial lymphocytes. The upper limit of IEL may be considered 20 or 25 according to the country, institution, and physician’s preference, although mostly 25 seems to be the most accepted current threshold.

**Table 2:** Corazza-Villanacci classification

Grade A
Nonatrophic, with normal V:C ratio and ↑ IELs (> 25 IELs/100 enterocytes)
Grade B1
"Atrophic", V:C < 3:1, but villi still detectable and ↑ IELs (> 25 IELs/100 enterocytes)
Grade B2
Atrophic and flat, villi not detectable and ↑ IELs (> 25 IELs per 100 enterocytes)

V:C: Villous to crypt ratio (normal, V:C > 3:1); GSE: Gluten-sensitive enteropathy; IEL: Intraepithelial lymphocytes.

Since the histological changes of GSE may be patchy in nature, a satisfactory number of biopsies need to be taken. It has been suggested that at least 4 distal duodenal biopsies and at least 2 biopsies of the duodenal bulb should be performed[61,65]. In consideration of the patchiness, mainly in the paediatric age, many institutions advocate for 6-8 distal duodenal biopsies and 2 biopsies of the duodenal bulb (CS, personal communication).

## IEL-IMMUNOPHENOTYPE

T cell receptors (TCRs) and surface co-receptors are used to characterize the immunological phenotype of the IELs. Normal duodenal biopsies should show in about 90% of healthy individuals a population of IELs which are CD3- and CD8-positive and mostly bearing TCRαβ. Conversely, CD4-positive T lymphocytes are few. In 1/10 of healthy individuals, a distinct population of TCRγδ-expressing lymphocytes has been recognized[39,66,67]. It is indeed an integrin of the β7 family, precisely CD103, which is responsible for the adhesion of the T lymphocytes to epithelial cells[68-70]. If frozen tissue is available, immunohistochemical typing for TCRγδ of T lymphocytes can be performed. In GSE, TCRγδ may reach up to 30%[71].

IEL is constituted mainly by CD8-positive CD3-positive lymphocytes representing the most sensitive immunohistochemical features of GSE. From 40 IELs to 25 or 20 IELs per 100 epithelial cells has been a long journey and immunohistochemistry may help, but may also lead to over-diagnosis of GSE[72]. This may be the case in infiltrative-type lesions in an individual patient with suspected GSE, where the duodenal biopsy fails to show an abnormal architecture and the IEL count is difficult to perceptively be assessed adequately. Such situation, although uncommon, may be a sign of latent GSE, despite other causes possibly being at the origin of this finding. To the best of our knowledge, its clinical relevance remains to be adequately assessed by long-term follow-up studies.

**Figure 2:**
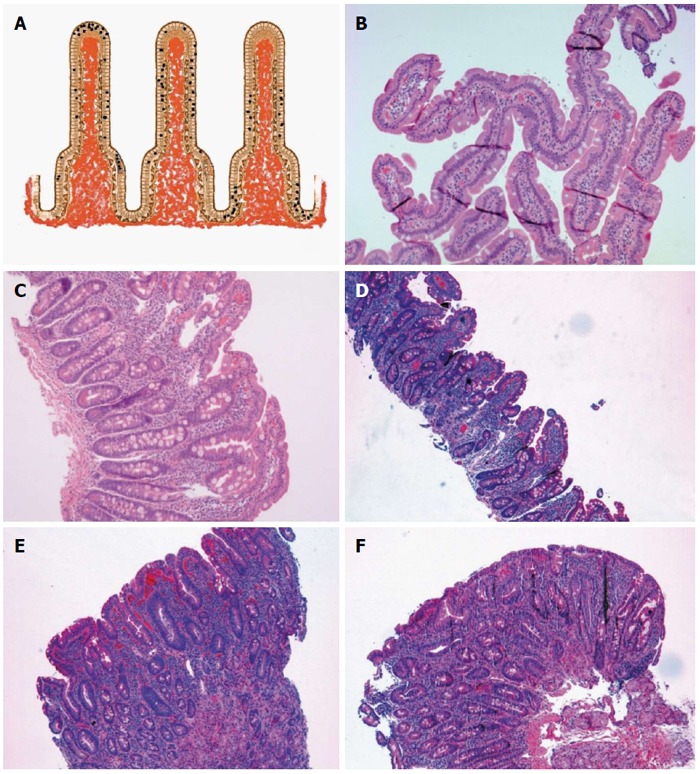
Intraepithelial lymphocytes and Marsh classification. A: Schema of the intraepithelial distribution of the intraepithelial lymphocytes (top, side and bottom, see text); B: Marsh 0, normal villous architecture with en-face cut (HE, × 100); C: Marsh I (HE, × 100); D: Marsh IIIA (HE, × 100); E: Marsh IIIB (HE, × 100); F: Marsh IIIC (HE, × 100). Marsh-Oberhuber classification is often shortened as Marsh.

An increased IEL count in an otherwise normal small bowel biopsy specimen is obviously not specific for GSE and may be associated with numerous conditions such as non-steroidal anti-inflammatory drugs (NSAIDs) use, microorganisms, bacterial overgrowth, immunological disorders, and lymphocytic or collagenous colitis among others. In examining the biopsies of patients with GSE, the number of CD8-positive CD3-positive T-lymphocytes is in crescendo towards the villous tips, while normal villi or non-GSE lymphocytes show a crescendo towards the base of the villi (crescendo *vs* decrescendo pattern) (Figure 2)[9,13,59]. Immunohistochemical investigation for TCRγδ in IEL is as sensitive and specific as the villous tip IEL count and may result in distinguishing other intestinal disorders from GSE in an effective way; but, to date, TCRγδ immunohistochemistry in early and latent GSE remains still controversial[24,73]. Moreover, the initial attempts to perform an assay using formalin-fixed and paraffin-embedded tissue blocks have been in vain[24].

Refractory gluten-sensitive enteropathy (RGSE) is a term used to define a pathological condition affecting the small bowel, histologically resembling GSE but not responding to a strict GFD of at least 6 mo[74]. In RGSE, most IELs have an abnormal immunophenotype, characterized by intracytoplasmic CD3ε and CD103 and loss of expression of CD3, CD4 or CD8 as well as TCR on the cell surface in 52%-98% of cases associated with a restricted rearrangement of the TCRγ gene[74,75]. In about 3/4 of patients with refractory sprue, clonal TCRγ gene rearrangement is seen and the CD3 T cell lymphocytes of the lamina propria are constituted by a mixture of both CD4 and CD8 T lymphocytes[67].

Type I RGSE is characterized by a normal T cell phenotype (CD3^+^/CD8^+^), while type II RGSE shows, by molecular investigations, loss of CD8 expression and clonality. Type II RGSE may progress to enteropathy-associated T cell lymphoma. In addition to the absolute number of IELs, the distribution of CD8-positive CD3-positive T lymphocytes along the villous has been observed to vary in GSE as well as in RGSE.

## GSE-MIMICKERS - “COMMON, LESS COMMON AND HIGHLY UNCOMMON”

GSE mimickers are defined as diseases that may mimic GSE leaving the patients to a wrong clinical management. The Latin poet Virgil (70-19 BC) wrote in his book of the Georgics of the 1^st^ century BC a quite famous sentence, ”*Felix, qui potuit rerum cognoscere causas*” (literally translated as: Privileged who was able to know the causes of things) that may be appropriate in this context. IELs alone may not be diagnostic of GSE, because there are many GSE mimickers. In determinate situations, the location of IELs may help. Top or apical IEL may be suggestive of GSE and particularly of latent GSE or GSE at early stage with preserved villous architecture.

IELs are more likely to decrease along the villous tip in non-GSE, laterally located and patchy distributed IEL may be seen in IBD, while low down cryptically located IEL may suggest graft *vs* host disease (GvHD) or allograft rejection in an appropriate clinical setting. Indeed, the initial manifestation of an IBD has been recorded in the duodenum, before changes occur in the terminal ileum or large bowel. Focal acute inflammation is defined by the presence of a cluster of more than one (> 1) neutrophilic granulocyte in the lamina propria or epithelium and more than one (> 1) focus in a tissue biopsy[76-78]. Some other authors suggest that neutrophilic granulocytes may be normal components of the lamina propria, provided no invasion of the crypt or surface epithelium is detected[29], but we do not agree because of the specific nature of this inflammatory cell.

Focal acute duodenitis is not a sensitive feature in Crohn’s disease, but has high specificity (92%) and high predictive value (93%-95%)[78]. Precursors of aphthoid ulcers may be considered foci of acute inflammation detected in the surface epithelium and deep stroma of the duodenum. The duodenum is also affected by acute inflammation with or without stomach involvement, but the incidence of granulomas is quite variable depending on the age of the patients and duration of the disease. The interobserver variability of interpreting duodenal biopsies may show different kappa factor depending from the institution[60,79].

**Figure 3:**
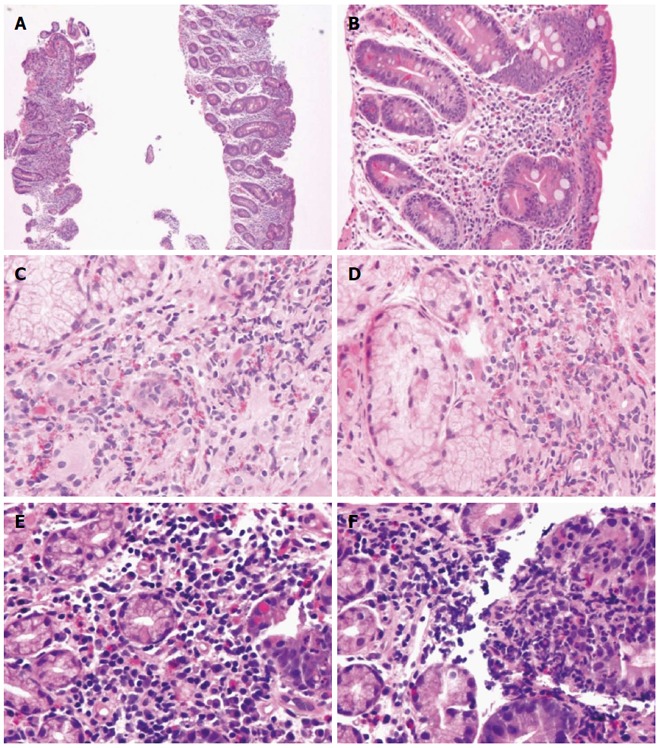
Gluten-sensitive enteropathy and GSE mimickers. A, B: Variable destructive patterns of Marsh IIIC of GSE (A: HE, × 100; B: HE, × 200); C, D: Eosinophilic duodenitis (HE, × 400); E, F: Peptic duodenitis (HE, × 400). GSE: Gluten-sensitive enteropathy.

**Figure 4:**
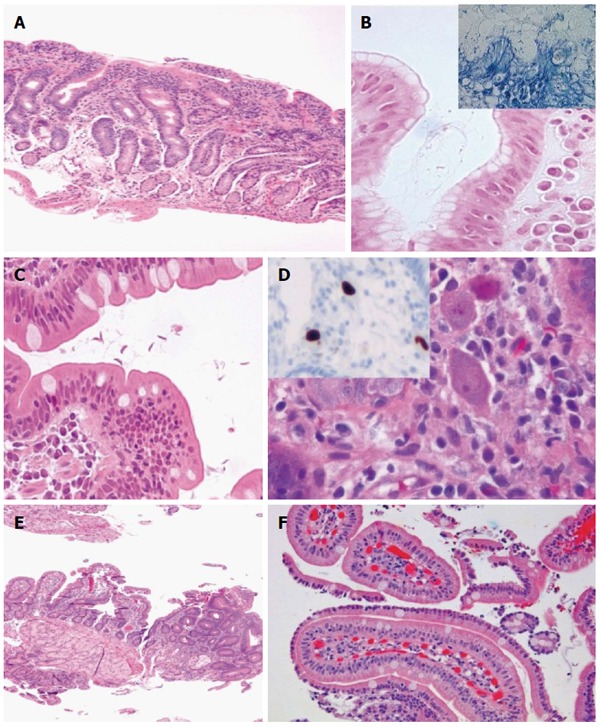
Gluten-sensitive enteropathy mimickers. A: Lymphocytic gastritis with involvement of the duodenum (HE, × 100); B: *H. pylori* gastritis (inset, Giemsa staining) (HE and Giemsa × 630); C: Giardiasis (HE, × 400); D: Cytomegalovirus (CMV) infection (HE, × 6300) and inset showing anti-CMV antibody reacting against viral proteins using an avidin-biotin complex immunoperoxidase immunohistochemical detection (× 100); E: Focal adenomatous change in duodenum (HE, × 50); F: Sickle cell disease-related duodenitis (HE, × 200).

**Figure 5:**
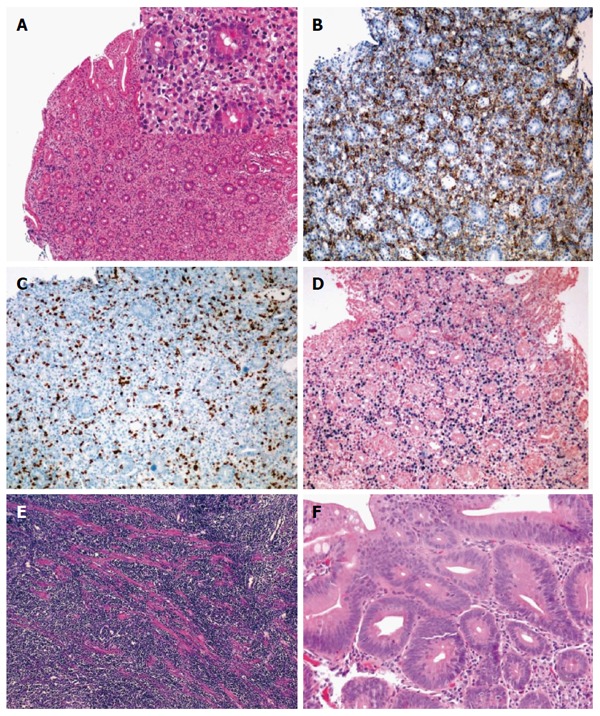
Gluten-sensitive enteropathy mimickers. A-D: Post-transplant lymphoproliferative disorder, polymorphic type (A: HE, × 50 and inset HE, × 200) showing mononuclear epithelial and stromal infiltration with blasts and high CD20 (B, × 100) on CD3 (C, × 100) lymphocytes and high Epstein-Barr virus replication (*in situ* hybridization or Epstein-Barr encoding region, × 100); E: Burkitt lymphoma of the duodenum (HE, × 100); F: Tubular adenoma of the duodenum of a patient with familiar adenomatous polyposis (HE, × 200).

**Figure 6:**
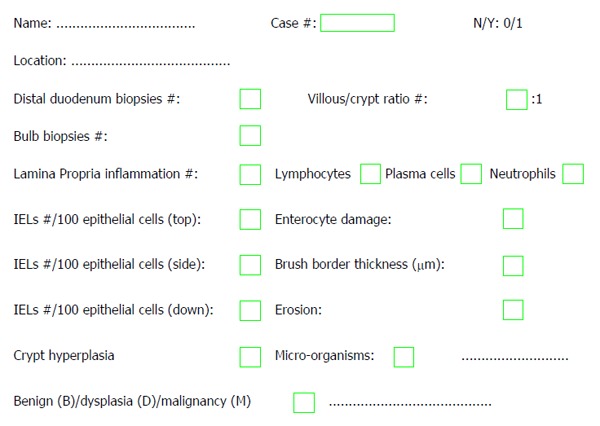
Synoptic report for gluten-sensitive enteropathy and gluten-sensitive enteropathy mimickers.

IEL distribution seems to be highly sensitive, but it may require additional training in the interpretation of the histology of the upper gastrointestinal tract. The diagnosis of GSE may remain problematic, because no single test shows 100% sensitivity and 100% specificity in every patient[12]. GSE mimickers may be indeed behind the scene, and there is undoubtedly no other field in gastroenterology better pictured by the Virgilian sentence (Figures 2, 3, 4, 5 and 6).

The three most common GSE mimickers are gastric *Helicobacter pylori* (*H. pylori*) infection, medications, especially NSAIDs or proton-pump inhibitors (PPIs), and IBD[80]. *H. pylori* infection is associated with chronic active gastritis, ulcer disease, chronic active duodenitis and bulbitis, while non-specific duodenitis or peptic duodenitis are conditions associated with acid injury. *H. pylori* infection is typically associated with duodenal gastric metaplasia, characterized by foci of gastric-type mucus-secreting cells interspersed between duodenal enterocytes, which may be easily recognized by the periodic acid Schiff (PAS)-positivity of the cells containing neutral mucin and the lack of the brush border[81,82]. An increased IEL count is observed in the duodenum of patients with *H. pylori* gastritis[9,15,83,84]. There is still some debate about the specificity of the findings and more longitudinal studies may be needed, but in any case, correlation with serology and gastric biopsies is still recommended.

The use of NSAIDs has been associated in a few cases of duodenal IEL[85,86]. Brunner gland hyperplasia, originally thought of as a feature of peptic duodenitis, is now considered not relevant, because it may be encountered in the normal duodenum as well[29]. Other medications associated with villous architectural changes include colchicine, mycofenolate mofetil, ipilimumab and several chemotherapy agents or radio-/chemotherapy protocols among others.

Both Crohn’s disease and ulcerative colitis may have IELs in the duodenum[76,87-90]. Duodenal granulomas are a very helpful finding in confirming the diagnosis of Crohn’s disease, but they are seen in less than half of the patients. In patients with classical presentation of Crohn’s disease, villous shortening accompanied by neutrophil-rich inflammation, oedema in the lamina propria and crypt abscesses may also be encountered. Crucial is the correlation with biopsy findings arising from other sites, because isolated duodenal Crohn’s disease is extremely rare[91].

In up to 1/4 of patients with ulcerative colitis, variable villous blunting, inflammatory expansion of the lamina propria by plasma cells and active (neutrophilic) inflammation are also seen[90,92,93]. Food allergy, infections, small intestine bacterial overgrowth, tropical sprue and various immunological or autoimmune disorders have been described, but are less common. Uncommon events with IEL and villous blunting may include sickle cell anaemia-duodenitis with a potential ischemic background due to pileup of abnormally shaped erythrocytes[94,95] and non-*H. pylori* infection, such as with *Yersinia enterocolitis* and *Salmonella spp*.[96,97].

Food allergy enteropathy (FAE) and cow’s milk protein-sensitive enteropathy (CMSE) may be encountered in a duodenal biopsy and both conditions may show a wide variety of mucosal lesions in any part of the upper and lower gut. Although villous atrophy is not seen in food allergy, crypt hyperplasia and an increased number of inflammatory cells, particularly eosinophilic granulocytes, are detected more often in the lamina propria and rarely also in the surface epithelium[32,33,98-103]. In CMSE, the villous architecture is usually normal, but cytotoxic IEL count is increased, particularly in the descending part of the duodenum in contrast to GSE, which conversely shows the most severe changes in the proximal parts of the duodenum[104-109].

Infections that are commonly seen in the duodenum include giardiasis, cryptosporidiosis, microsporidiosis, cyclosporiasis, isosporiasis, Whipple’s disease, *Mycobacterium avium intracellulare*, visceral leishmaniasis, cryptococcosis and cytomegalovirus. The clinical history with the most recent travel history of both parents and children, the geographical settings and the age of the patient may aim to restrict the diagnosis that needs to be confirmed by the laboratory and microbiologic analysis. Morphologically, *Giardia lamblia* is a pear-shaped microorganism if cut lengthwise and a binucleate, ventral disc if cut frontally, with 4 pairs of flagella. *Cryptosporidia* are basophilic merozoites (2-5 μm) of varying size. *Microsporidia* are supranuclear parasitophorous vacuoles indenting the nucleus. *Cyclospora* are round and fusiform merozoites (up to 6 μm in length) with supra-nuclear parasitephorous vacuoles. *Isosporas* are subnuclear parasitophorous vacuoles (20-30 μm) containing banana-shaped merozoites and sexual forms. *Tropheryma whipplei* are PAS-positive bacilli. I*ntracellular M. avia* are acid fast and diastase-resistant (D-)PAS-positive curved bacilli. *L. donovani* are 1-2 μm basophilic amastigotes in an identifiable parasitophorous vacuole. Cryptococci are microorganisms with narrow-neck budding, while the characteristic aspect of cytomegalovirus is the “owl’s eye”, which displays a dense nuclear inclusion and granular cytoplasmic inclusions. In Table 3, the differential diagnosis of these microorganisms, with the histological changes observed in the duodenum, are summarized.

**Table 3:** Most common duodenal infections potentially mimicking gluten-sensitive enteropathy

	**Agent**	**Villi**	**CH**	**LPI**	**IELs**	**PMNs**	**Sup.-D**.	**Target**	**Site**	**Stain**
Giardiasis	*G. lamblia*	0-3A	Nil	0/+/++	< 20/↑	Rarely	Nil	None	IL	GS
Cryptosporidia	*C. parvum*	1-3A	+	+/++	< 20	Focal	Focally	Enterocytes	IE	WS
Microsporidia	*E. bieneusi*	1-3A	+	+/++	< 20	Nil	Focally	Enterocytes/macrophages	IEv/ IEc	WS
	*E. intestinalis*									
Cyclospora	*C. coyetanensis*	1-3A	+	+/++	< 20/↑	Nil	Focally	Enterocytes	IEv	NA
Isospora	*I. belli*	1-3A	+	+/++	< 20	Nil	Focally	Enterocytes	IEv	NA
Whipple D	*T. whipplei*	1-3A	Nil	++^1^	↑	Nil	Nil	Macrophages	LP	PAS
										ZN
MAI	*M. avium intracellulare*	1-3A	Nil	++^1^	< 20	Nil	Nil	Macrophages	LP	PAS
										ZN
										AR
Leishmaniasis	*L. donovani*	1-3A	Nil	+/++	< 20	Nil	Nil	Macrophages	LP	NA
Cryptococcosis	*C. neoformans*	1-3A	Nil	0/+	< 20	Nil	Nil	None	LP	DPAS
										MS
CMV	*Cytomegalovirus*	Ulcers	2	+/++	< 20/↑	+/-	Focally	Epithelium/endothelium	LP	IHC

1 Pale macrophages;

2 Crypt damage; IIIA partial atrophy, IIIB subtotal atrophy, IIIC total atrophy. 0-3A: 0, no inflammation, 1, IELs, 2 mild hyperplasia/mild inflammation. IL: Intraluminal; IE: Intraepithelial (surface and crypt epithelium); IEv: Intraepithelial at the villous tips; IEc: Intraepithelial at the crypts; LP: Lamina propria; CH: Crypt hyperplasia; AR: Auramine-rhodamine stain; DPAS: Diastase-periodic acid Schiff stain; GS: Giemsa stain; IHC: Immunohistochemistry with antibodies against the cytomegalovirus antigens; MS: Methenamine-silver stain; WS: Warthin-Starry stain; ZN: Ziehl-Nielsen stain.

Autoimmune enteropathy (AIE) is another GSE-mimicker because it is characterized by villous atrophy unresponsive to a GFD[25,110]. The histological evidence of enteropathy, a lack of any triggering food protein, anti-enterocyte antibodies as well as persistent diarrhoea after prolonged fasting and presence of organ-specific serum antibodies are essential for the diagnosis of this entity. The histology of AIE is characterized by variable degrees of architectural changes, including normal to total villous atrophy and a CD8-predominant immunophenotype of IELs. IEL count may be normal or increased and is mainly characterized by CD8-positive lymphocytes. Importantly, the number of lymphocytes harbouring γδ immunophenotype is normal in both surface epithelium and lamina propria help in distinguishing AIE from GSE. AIE may produce subtotal villous blunting and IEL simulating the appearance of GSE, but the absence of goblet cells and Paneth cells in AIE biopsies accompanied by a prominent crypt apoptosis are helpful clues[29].

Common variable immunodeficiency (CVID) may also manifest with gastrointestinal symptoms, being the second most common primary immunodeficiency, and its diagnosis relies on recurrent infections, decreased IgG levels at least 2-standard deviations below normal with at least decreased levels of one other immunoglobulin subclass, exclusion of other causes of immunodeficiency, and a failure to mount a response to vaccination. In 2/3 patients with CVID undergoing endoscopy, the duodenal biopsy shows IEL with or without villous architectural changes and 2 CVID characteristic clues are the paucity or absence of plasma cells with prominent crypt apoptosis in CVID[29,111-113].

GvHD and allograft bowel rejection (AGBR) may be ruled out on clinical settings. GvHD may, however, come to the gastroenterologist or pathologist who are not provided with the history of bone marrow transplantation for instance. GvHD may have, although uncommonly, an increased IEL count in proximal small bowel biopsies. A decrescendo from base to apical villi and the finding of epithelial cell apoptosis in the deep crypts, with or without some degree of architectural disturbance, together with the clinical setting may help to address this diagnosis[114].

Collagenous sprue (COS) is an GSE mimicker, originally described by Weinstein in 1970[115], and shares several aspects of GSE, including villous architectural abnormalities, IEL and crypt hyperplasia; but, an irregularly thickened layer of type 1 collagen just subjacent to the surface epithelium is extremely useful for distinguishing COS from GSE. A monotypic, truncated immunoglobulin α heavy chain lacking an associated light chain secreted by plasma cells infiltrating the bowel wall characterizes a condition called immunoproliferative small intestine disease (IPSID), which is a MALT lymphoma[116-118]. The early stages of IPSID may be quite challenging, because the duodenal mucosa may appear normal or near-normal, but thickening, erythema and nodularity of the mucosal folds may be observed in the duodenum and upper jejunum at later stages[117]. IPSID is mostly reported in individuals from the Middle East, North and South Africa and the Far East, and the epidemiological background may be quite helpful.

Several immune-related disorders, including Hashimoto thyroiditis, Graves’ disease, rheumatoid arthritis, psoriasis and systemic lupus erythematosus may cause IEL. These diseases need to be carefully ruled out on both clinical and laboratory grounds. Other etiologic factors that have been associated with IEL are quite more uncommon and infrequently associated to IEL, but they also need clinical and laboratory correlation. These disorders involve the nervous system mainly, the two major diseases of which include autism and multiple sclerosis[119,120].

The enteropathy-type intestinal T cell lymphoma (EITL) may be considered a complication of long-standing GSE[121-123]. EITL is frequently multifocal with ulcerative lesions and a tendency to perforate either at presentation or during chemotherapy. Histologically, there is a pleomorphic medium-to-large cell population constituted by the expression of CD3 and lack of CD4 and CD8 expressions as well as a small and monomorphic cell population characterized by the expression of CD3, CD8 and CD56 and lack of CD4 expression. CD30 is always present in the tumour cells and may be seen in the adjacent villi of the lymphoma lesions, and is considered an ominous marker for prognosis. Among the neoplastic GSE mimickers, the tubular adenomas, post-transplant lymphoproliferative disorders (PTLDs) and lymphomas should be listed.

## GSE - CHALLENGES ACROSS OCEANS

In 2012[16], the guidelines for GSE diagnosis were issued by the ESPGHAN suggesting that biopsies can be avoided in patients who have positive HLA-DQ test results. It has been suggested that HLA-DQ test may extend beyond these cases[41]. The main inhibitions in efficiently using molecular biology techniques are represented by cost and lack of automation, but RT-PCR, digital PCR and next-generation sequencing may today open interesting possibilities in tailoring the diagnostic algorithm for GSE in a more efficient way. The combined use of aTTG and anti-DGP assay is now recommended in young children, while HLA-DQ typing is useful in support of histology in seronegative patients, and to exclude patients at high risk for GSE. In patients with low risk for development of GSE, the presence of IgA aTTG-positive blood inclines towards endoscopy and duodenal biopsy. ESPGHAN emphasizes that patients with selective IgA deficiency should be tested for anti-DGP IgG and/or aTTG IgG and, if positive, a biopsy needs to be performed.

The guidelines of the American College of Gastroenterology and World Gastroenterology Organization are similar, but differences have been identified recently[41]. These two guidelines distinguish between patients at low and high risk of GSE and in screening the general population, with a GSE prevalence of 1%, the IgA aTTG and DGP assays are now recommended, either simultaneously, or in sequence. Thus, in the high-risk population, only one test is considered sufficient, because in these patients it is supposed that additional tests do not increase the reliability of screening results. Conversely, in low-risk patients, a positive serological test is a strong indication for duodenal biopsy that remains the gold standard in North America. For patients at risk of GSE, biopsy is always recommended, irrespective of serological results; if the results of both tests are positive, the diagnosis of GSE is confirmed. Conversely, if the serology is positive and histology is negative, it has been suggested that the biopsy is repeated at least after 1 year. If the histology is positive and serology is negative, HLA-DQ typing is counselled and other possible causes of duodenitis should be carefully evaluated. GSE is ruled out only when both serology and histology are negative. In Table 4, the main differences between the GSE guidelines across oceans are presented[41].

**Table 4:** North American - European divergences across oceans in gluten-sensitive enteropathy

	**Target**	**Screening**	**PS Tests^1^**	**HLA-DQ**	**EMA**	**AGA**
ESPGHAN	Paediatric	Anti-tTG-IgA and IgA^2^	Anti-tTG-IgG/anti-DPG-IgG^3^	Yes, if	Yes, in confirming PS tests	No
				↑EMA/anti-tTG		
ACG	Paediatric/adult	Anti-tTG-IgA	Anti-tTG-IgG/anti-DPG-IgG^3^	Yes, if biopsy/serology disagreement	NS	No
WGO	NS	Anti-tTG-IgA/anti-DPG IgG	NS	Yes, if biopsy/serology disagreement	Yes, in confirming PS tests	No

1 Postscreening tests;

2 Total serum IgA;

3 Anti-DPG: Anti-deamidated gliadin peptide. ESPGHA: European Society of Pediatric Gastroenterology, Hepatology and Nutrition; ACG: American College of Gastroenterology; WGO: World Gastroenterology Organization; NS: Not specified.

## DUODENAL MUCOSA - SYNOPTIC REPORT

An integrated assessment of the histopathology elementary lesions and clinical and serological findings make consistent and reliable the diagnosis of GSE. The elementary lesions consist of (1) *increased IELs* or IEL with a value between 20 and 24 IEL/100 enterocytes as borderline and ≥ 25 IEL/100 enterocytes representing a pathological lymphocytic infiltration of the surface epithelium; (2) *decreased height of the enterocytes* with flattening of enterocytes, intracytoplasmic vacuolation as well as reduction or absence of brush-border; (3) *crypt hyperplasia* as indicated by extension of the regenerative epithelial crypts associated with changes in the presence of more than 1 mitosis per crypt; and (4) *villous blunting* identified as decrease in villous height, alteration of normal crypt/villous ratio (3:1) until total disappearance of villi with proper orientation of the biopsies[124].

A synoptic report is commonly used for cancer pathologies, using checklists that allow a better management of patients with oncological disease[125,126]. Free text reports often demonstrate significantly impaired data collection when recording several parameters, and the number of words used is also significantly reduced using pre-formatted structured reports as compared to free text reports. In public healthcare, the introduction of a structured reporting dictation template improves data collection remarkably and reduces the subsequent administrative burden when dealing with phone calls and/or reviewing the number of cases reviewed at multidisciplinary team meetings, and external quality assurance programs provide a support for it[127].

In our opinion, a biopsy report should include the number and site of the biopsy specimens, the pathology or normality of the tissue specimens, the villous-crypt ratio, the villous architecture (normal or blunted, partial/total), the IEL counts at the top, side and bottom, the morphology of the surface enterocytes (normal, flattened or damaged) with or without preservation or loss of the brush border, crypt hyperplasia, gastric metaplasia (*e.g*., chronic duodenitis), presence of microorganisms (*e.g., Giardia lamblia*, cryptosporidia, microsporidia, *Isospora belli*, cyclospora, *Mycobacterium avium intracellulare*, cytomegalovirus, *Cryptococcus neoformans*)[24]. A number of additional features have been suggested to be present in the histopathologic report[71,128], including the search results for potential benign, dysplastic or neoplastic lesions (*e.g*., adenoma or carcinoma, carcinoid, lymphoma). Figure 6 displays a synoptic report that may be considered useful for both clinics and research.

## CONCLUSIONS AND FUTURE PERSPECTIVES

Partial and patchy villous blunting may be found in CMSE, in postenteritis enteropathy and in GSE. Thus, multiple biopsies should be taken to minimise the risk of misdiagnosis. The bulb mucosa may be the only duodenal area affected and total or moderate villous atrophy may affect the duodenal bulb exclusively with a normal distal duodenum. Therefore, careful appreciation with regard to whether specimens are taken from the bulb or the descending part of the duodenum is essential[129]. GSE is a common cause of an increased IEL count in the duodenum accounting, probably, for up half of the cases, but GSE mimickers should be taken into account. New molecular biology-supported methodologies may tailor and individualize the diagnostic algorithm in the future.
